# Pollen from the Deep-Sea: A Breakthrough in the Mystery of the Ice Ages

**DOI:** 10.3389/fpls.2018.00038

**Published:** 2018-01-26

**Authors:** María F. Sánchez Goñi, Stéphanie Desprat, William J. Fletcher, César Morales-Molino, Filipa Naughton, Dulce Oliveira, Dunia H. Urrego, Coralie Zorzi

**Affiliations:** ^1^École Pratique des Hautes Études, EPHE PSL University, Paris, France; ^2^Environnements et Paléoenvironnements Océaniques et Continentaux, UMR 5805, Université de Bordeaux, Pessac, France; ^3^Quaternary Environments and Geoarchaeology, Department of Geography, School of Environment, Education and Development, The University of Manchester, Manchester, United Kingdom; ^4^Instituto Português do Mar e da Atmosfera, Portuguese Institute of Sea and Atmosphere, Lisbon, Portugal; ^5^Center of Marine Sciences, Algarve University, Faro, Portugal; ^6^College of Life and Environmental Sciences, University of Exeter, Exeter, United Kingdom; ^7^GEOTOP, Université du Québec à Montréal, Montreal, QC, Canada

**Keywords:** vegetation, millennial-scale climate variability, Dansgaard-Oeschger cycles, Heinrich events, glaciations, interglacials, Europe, Quaternary

## Abstract

Pollen from deep-sea sedimentary sequences provides an integrated regional reconstruction of vegetation and climate (temperature, precipitation, and seasonality) on the adjacent continent. More importantly, the direct correlation of pollen, marine and ice indicators allows comparison of the atmospheric climatic changes that have affected the continent with the response of the Earth’s other reservoirs, i.e., the oceans and cryosphere, without any chronological uncertainty. The study of long continuous pollen records from the European margin has revealed a changing and complex interplay between European climate, North Atlantic sea surface temperatures (SSTs), ice growth and decay, and high- and low-latitude forcing at orbital and millennial timescales. These records have shown that the amplitude of the last five terrestrial interglacials was similar above 40°N, while below 40°N their magnitude differed due to precession-modulated changes in seasonality and, particularly, winter precipitation. These records also showed that vegetation response was in dynamic equilibrium with rapid climate changes such as the Dangaard-Oeschger (D-O) cycles and Heinrich events, similar in magnitude and velocity to the ongoing global warming. However, the magnitude of the millennial-scale warming events of the last glacial period was regionally-specific. Precession seems to have imprinted regions below 40°N while obliquity, which controls average annual temperature, probably mediated the impact of D-O warming events above 40°N. A decoupling between high- and low-latitude climate was also observed within last glacial warm (Greenland interstadials) and cold phases (Greenland stadials). The synchronous response of western European vegetation/climate and eastern North Atlantic SSTs to D-O cycles was not a pervasive feature throughout the Quaternary. During periods of ice growth such as MIS 5a/4, MIS 11c/b and MIS 19c/b, repeated millennial-scale cold-air/warm-sea decoupling events occurred on the European margin superimposed to a long-term air-sea decoupling trend. Strong air-sea thermal contrasts promoted the production of water vapor that was then transported northward by the westerlies and fed ice sheets. This interaction between long-term and shorter time-scale climatic variability may have amplified insolation decreases and thus explain the Ice Ages. This hypothesis should be tested by the integration of stochastic processes in Earth models of intermediate complexity.

## Introduction

“To look in the oceans for direct evidence of past continental climates seems paradoxical. However, marine sediments contain far better terrestrial paleoclimate records than most continental deposits (Heusser, 1986/1987).” This visionary statement, based on previous seminal works published in the 1960s in a special issue of Marine Geology on Marine Palynology (e.g., Groot and Groot, 1966; Traverse and Ginsburg, 1966; Zagwijn and Veenstra, 1966), anticipated the major contribution that pollen analysis from marine cores would make to Paleoclimatology in subsequent decades. It is now well-established that long and continuous deep-sea sedimentary sequences collected near the continents provide high-quality and chronologically well-constrained pollen records documenting past changes in the vegetation and climate of the adjacent landmasses. Since the pioneering work of Heusser, Hooghiemstra, Rossignol-Strick, Van Campo, and Turon in the 1970 and 1980s in the North Pacific and the Atlantic Oceans and the Mediterranean and Arabian Seas (for references see **Supplementary Table S1**), an increasing number of deep-sea pollen records has been published from the Iberian and South African margins, the Mediterranean Sea, and the South Pacific and Indian Oceans. They mainly span all or a part of the last 1 million years. These records, which compare without chronological ambiguity marine and terrestrial stratigraphies, are pivotal for documenting potential leads and lags between regional atmospherically-driven vegetation, oceanic conditions and ice dynamics. The relevance of this approach was shown in two seminal papers where the authors demonstrated that the Eemian interglacial, ca. 128,000 years ago (128 ka), was not exactly the terrestrial equivalent of the marine interglacial Marine Isotope Stage (MIS) 5e, and that warmth and forest persisted in south-western Europe during periods of Northern Hemisphere ice growth (Sánchez Goñi et al., 1999; Shackleton et al., 2003). Therefore, pollen-rich marine records are of prime importance for understanding the interactions between the ocean and the atmosphere leading to the orbitally-paced deglaciations and glacial inceptions. More recently, coupled analysis of pollen and marine climatic tracers at a finer resolution has revealed a unique suitability to investigate the timing and amplitude of rapid millennial-scale climate changes in different regions of the world as well (e.g., Sánchez Goñi et al., 2008). This direct sea-land comparison approach also allows testing whether vegetation response to short periods of forcing is in dynamic equilibrium with climate (Webb, 1986) or if there is a lag between climate change and vegetation response (disequilibrium hypothesis, Birks, 1981; Bennett and Willis, 1995), due to species competition or different migration rates.

In the first part of the paper, we focus on the suitability of pollen analysis in deep-sea sediments to trace vegetation and atmospheric conditions through time, highlighting its key role in understanding the mechanisms underlying climate change via the direct comparison with ocean and ice dynamics. We also present a global compilation of pollen records obtained from Quaternary deep-sea cores, and identify the regions where few marine pollen records exist despite their relevance for the study of climate variability. The second part is devoted to the responses of the European vegetation and climate to long-term and, particularly, millennial-scale climate changes. We focus on western Europe because this region is directly affected by North Atlantic millennial-scale iceberg discharges, atmospheric Greenland warming and cooling events (Shackleton et al., 2000), and the close influence of the Northern Hemisphere ice growth and decay, as well as being strongly implicated in the feedback processes bringing the Earth’s system to glaciations (Ruddiman and Mcintyre, 1984). The third part of the paper aims to show that the interactions between short-term and long-term climate variability may be the potential missing piece of the Ice Age puzzle (Hodell, 2016), given that the Milankovitch astronomical theory of climate, i.e., changes in the seasonal distribution of the solar energy, alone cannot explain the ice age cycles (Milankovitch, 1941).

## Understanding Past Climate Changes

Climate is the sum of meteorological phenomena that characterizes the mean state of the atmosphere (i.e., temperature, precipitation, greenhouse gasses – GHG) over 30 years that depends, in turn, on the dynamics of various components of the climate system (Claussen, 2007). Insolation is the external astronomical (orbital) forcing that determines Earth’s regional climates and is defined as the amount of energy per surface unit that the Earth receives from the Sun. Insolation is controlled by the distance between the Earth and the Sun that depends on eccentricity (the shape of the Earth’s orbit), obliquity (the tilt of the Earth’s axis) and precession (the orientation of the Earth’s axis); the latter determines the amplitude of the seasons. These orbital parameters vary over time and trigger major climate changes with cyclicities of 100,000, 40,000, and 21,000 years (e.g., Berger and Loutre, 2004). A change in insolation affects the Earth’s five main climatic reservoirs-atmosphere, ocean, land surfaces, cryosphere, and vegetation- and each of them affects, in turn, the Earth’s other reservoirs through feedback mechanisms that amplify or reduce the original climate change (Ruddiman, 2001). Thus the frequency, duration and magnitude of a given climate change is the result of the interactions between orbital external forcing and internal feedback loops involving, for example, ocean currents, particularly the Atlantic Meridional Overturning Circulation (AMOC), ice-sheet and sea-ice dynamics, vegetation, volcanic eruptions, GHG concentrations, and albedo (Ruddiman, 2001).

**FIGURE 1:**
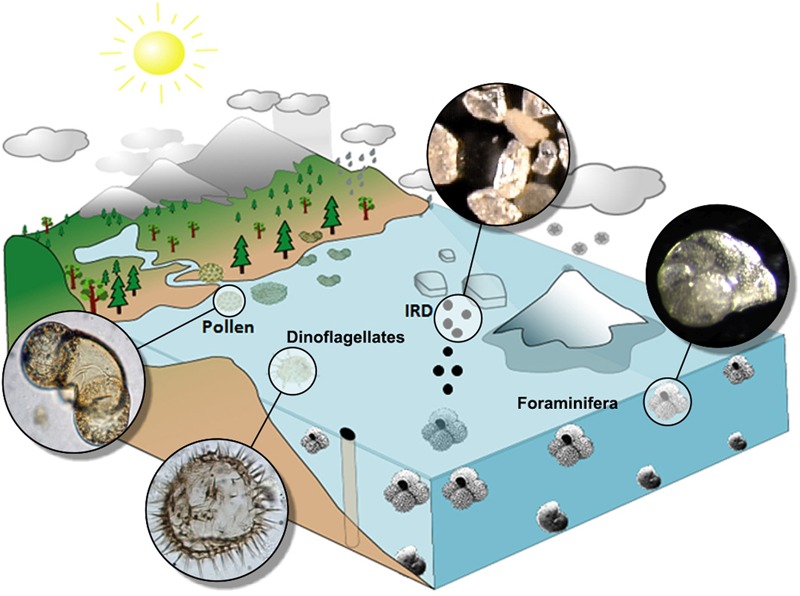
Illustration of a sediment core from a continental margin, which captures multiple terrestrial and oceanic tracers, including: transport and deposition of pollen grains, coarse sediments derived from the discharge and melting of icebergs [ice-rafted detritus (IRD)], planktonic (near surface) and benthic (bottom-dwelling) foraminifer, and dinoflagellates. Adapted from Sánchez Goñi (2016).

Marine and terrestrial palaeoclimatic records and ice archives allow the reconstruction of past climate changes in the Earth’s different reservoirs. However, understanding the mechanisms controlling the frequency, duration and amplitude of climate changes requires the comparison of these different records on a common timescale, a challenging task due to the fragmentary nature of certain sequences and the uncertainties inherent in different dating methods and age models used. The age models of marine and terrestrial cores are mostly based on radiometric dates (^14^C, U/Th…) and isotopic stratigraphy/orbital tuning, while the ice archives are dated by mean of layer counting or physical models of ice accumulation. The three types of records have uncertainties ranging from a few decades to several centuries and even millennia (e.g., Bazin et al., 2013). Continuous pollen-rich deep-sea sedimentary sequences trace the regional vegetation history and, consequently, the climate of the landmasses nearby and represent a unique way to circumvent the aforementioned issues when comparing different records (vegetation/atmosphere, marine, and ice). Marine climatic indicators from these sequences allow the quantitative reconstruction of sea surface temperature (SST) and salinity (e.g., planktonic foraminifera, dinoflagellate cysts, calcareous nannofossil and diatom assemblages, alkenones), deep ocean conditions (e.g., Mg/Ca, benthic foraminifer assemblages, carbon isotopic ratio -δ^13^C- of benthic foraminifera, Pa/Th), iceberg dynamics (Ice Rafted Debris or IRD, the coarse sediments transported by icebergs) and the ice volume stored in the ice caps [oxygen isotopic ratio -δ^18^O- of benthic foraminifera; although a bias exists between this isotopic ratio and global ice volume (Skinner and Shackleton, 2006)] (**Figure 1**). This approach is usually named “direct land-sea correlation or comparison,” although the insights extend not only to terrestrial and marine conditions but also to the cryosphere. Other indicators found in marine sediments give complementary information on terrestrial climate such as charcoal particles providing relevant information about climatically-driven fire regime prior the beginning of human impact on the environment (Daniau et al., 2007, 2013), dust concentrations (e.g., deMenocal, 2004; Jullien et al., 2007) and terrestrial organic compounds or biomarkers (δD, GDGT, δ^13^C from leaf waxes) that give useful information on precipitation and, in some cases, on temperatures (e.g., Weijers et al., 2007).

This direct correlation allows the identification of possible synchronicity or time-lags between the response of these reservoirs to a given climate change and, therefore, provides insights into the feedback processes underlying this climate change. Finally, the comparison between directly correlated palaeoclimatic data and model simulations allows the assessment of the reliability of the mechanisms implicated in the different models to reproduce the observed climate change (e.g., Sánchez Goñi et al., 2012). However, before identifying regional land-sea linkages associated with past climate changes, it is crucial to understand the present-day regional pollen signals.

### Pollen in Deep-Sea Sediments

A substantial quantity of pollen grains produced, released and dispersed from terrestrial higher plants (as well as spores of lower plants) reaches the ocean mainly by fluvial and atmospheric transport processes, with oceanic currents playing a negligible role (Koreneva, 1966; Stanley, 1966). The relative contribution of one or other transport vector is regionally-dependent (Dupont, 2011). In the Arctic, for instance, pollen is also transported by sea ice scouring and sediment transport in addition to northeasterly winds (Mudie and McCarthy, 2006). Marine sediments located further than 300 km offshore are weakly influenced by rivers and the dominant pollen transport is aeolian (e.g., Hooghiemstra et al., 1988). In contrast, the pollen preserved in marine sediments located under the influence of river plumes (nepheloid layers) is mainly of fluvial origin (e.g., Heusser and Balsam, 1977; Cambon et al., 1997; Mudie and McCarthy, 2006). Once it arrives in ocean surface waters, pollen is ingested by planktonic organisms and later integrated into their fecal pellets or agglomerated with clays (Mudie and McCarthy, 2006; Beaudouin et al., 2007). Due to these processes, pollen buoyancy decreases and is little influenced by ocean currents. It thus becomes an integral part of marine snow and crosses the water column with a relatively high speed (estimated at ∼100 m/day in the Atlantic water column) before being deposited at the bottom of the oceans (Hooghiemstra et al., 1992). Furthermore, sediments under the influence of upwelling are particularly rich in pollen for two reasons: the intensification of the downward particle transport through the water column (Ratmeyer et al., 1999) and the better preservation in almost anoxic sediments (Dupont, 2011).

**FIGURE 2:**
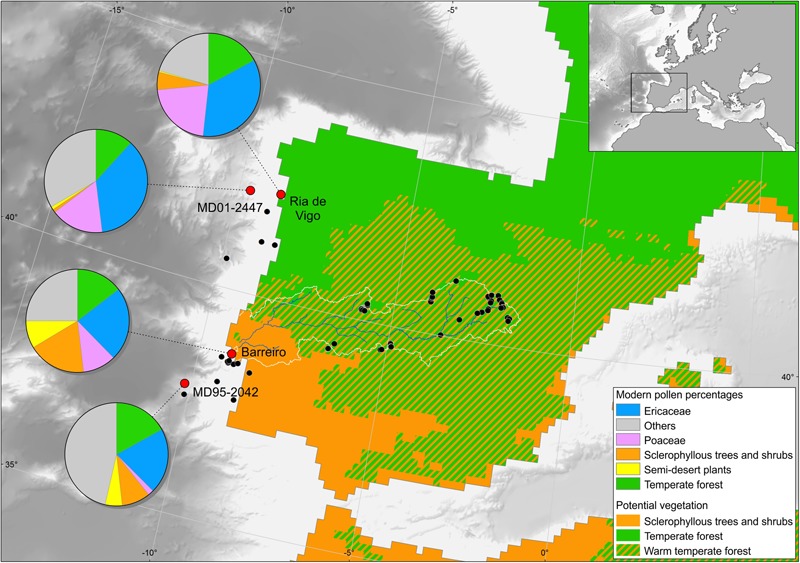
Map of the Iberian Peninsula with the main types of potential vegetation (Braun-Blanquet and Walter in Blanco Castro et al., 1997), present-day pollen signature in surface sedimentary samples from the Ría de Vigo and Barreiro (Tajo estuary), and samples from the top samples of cores MD99-2331 and MD95-2042 covering the last centuries and located in the Atlantic and Mediterranean region, respectively.

Pollen studies on modern deep ocean surface sediments from the Iberian margin (Turon, 1984a; Naughton et al., 2007), the Bay of Biscay (Turon, 1984b), the Mediterranean (Koreneva, 1971; Beaudouin et al., 2007), the African margin (Hooghmiestra et al., 1986; Dupont and Wyputta, 2003), and the western and eastern margins of North America and the Gulf of Mexico (Heusser and Balsam, 1977; Heusser, 1985; Heusser and Van de Geer, 1994) showed that pollen assemblages from the sediment surface of the ocean floor reflect an integrated image of the regional vegetation of the adjacent continent and, consequently, the climatic parameters under which this vegetation has developed. Palynological richness, i.e., the number of pollen types per sample standardized to a constant pollen sum, is similar in fossil samples from deep-sea cores, modern terrestrial and deep-sea surface samples, and fossil assemblages from lakes and estuaries, oscillating between 15 and 25 morphotypes with respect to a pollen sum of at least 100 terrestrial pollen grains (e.g., Sánchez Goñi and Hannon, 1999; Naughton et al., 2007). An ongoing study on the pollen representation of modern vegetation in moss-polsters along the Tajo basin shows that palynological richness ranges from 13 to 41 with respect to a pollen sum of 500 terrestrial pollen grains. In particular, Naughton et al. (2007) demonstrated by comparison of modern pollen spectra from the deep ocean, estuaries and continent that the pollen signal of the Iberian margin is similar to that found in estuarine sediments from western Iberia that reliably represents the broad regional vegetation of the related hydrographic basins (**Figure 2**). On the one hand, pollen assemblages from the northern part of the margin reflect the composition of temperate forest that develops in northwestern Iberia dominated by deciduous oaks. On the other hand, pollen samples from the southern part of the margin capture the composition of Mediterranean forest, i.e., warm-temperate forest dominated by sclerophyllous trees and shrubs that characterizes southern Iberia (**Figure 2**). In this region, pollen is mainly transported by the rivers because the dominant winds come from the north-west and the hydrographic Iberian basins are large and thus favor the transport of sediment load, including pollen (Dupont and Wyputta, 2003; Naughton et al., 2007). From a botanist’s point of view, this type of study has therefore a limitation for reconstruction of ancient local plant communities (Groot and Groot, 1966). However, the similarity of western European terrestrial pollen sequences and eastern North Atlantic deep-sea pollen records (e.g., Naughton et al., 2007 for the last 18,000 years in north-west Iberia; Fletcher and Sánchez Goñi, 2008 for the last 50,000 years in south-east Iberia; Sánchez Goñi et al., 2012 for the Last Interglacial in western France) demonstrate once more that marine pollen records provide a reliable image of the vegetation history of the adjacent landmasses (Groot and Groot, 1966). Moreover, the regional vegetation is directly linked to climate conditions as the present-day distribution of the major biomes is governed by climatic parameters (Bailey, 1998). For western Europe Gouveia et al. (2008) have recently shown the rapidity with which vegetation in this region responds to North Atlantic atmospheric processes, i.e., the westerlies.

The comparison between terrestrial and marine modern pollen samples to better interpret marine pollen records has been further improved and applied to south-western Africa (Urrego et al., 2015). In particular, modern pollen spectra were used to assess the distribution of Poaceae pollen abundance and other pollen taxa with potential indicator value for large biomes in southern Africa, and therefore climatic zones in this region. For instance, pollen percentages of Poaceae up to 70% reflect the Nama-karoo and its transition with the fined-leaved savanna. These results can substantially change the interpretation of the marine pollen fossil record collected off northwestern South Africa. Contrary to the studies that interpret the increase of Poaceae pollen percentages as the result of humidity-driven savanna expansions in southwestern Africa, the new pollen calibration study offers an alternative interpretation indicating that fine-leaved savanna developed in this region due to the aridity increase during the warm and humid Northern Hemisphere periods of the last interglacial (Urrego et al., 2015). This approach should be applied to other regions to improve the interpretation of marine pollen assemblages in terms of vegetation cover and composition. However, the quantification of the vegetation abundance from marine pollen assemblages to infer, among others, changes in albedo and their influence on climate change, remains a challenge. The REVEALS model was conceived to obtain quantitative reconstructions of regional vegetation cover around large lakes from pollen data, which resembles the spatial scale of marine pollen samples (Sugita, 2007). Nevertheless, this model only considers atmospheric pollen dispersal and deposition, thus disregarding the importance of pollen input from inlet streams and surface run-off, which are often the main vectors delivering pollen to the marine environment (Sugita, 2007).

**FIGURE 3:**
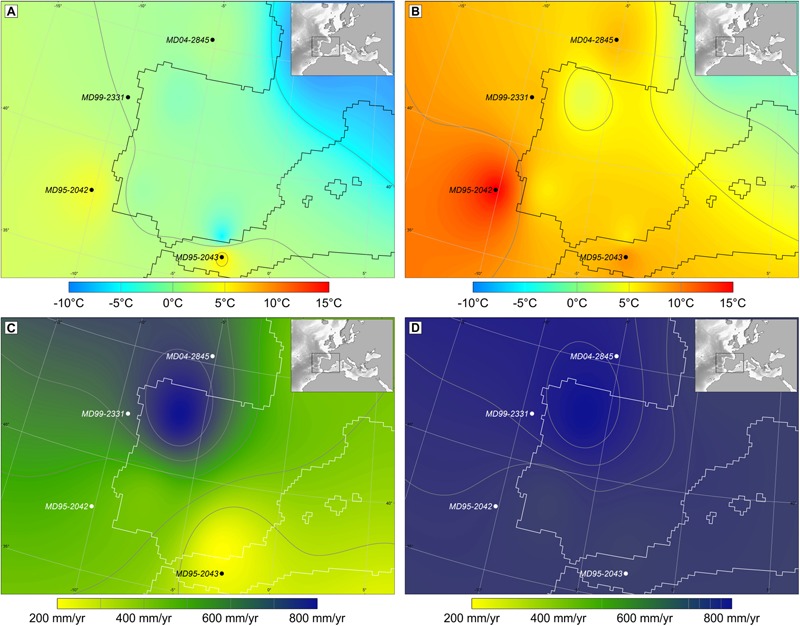
Maps of western Europe with pollen-based quantitative climate estimates, winter temperature and annual precipitation, and sea surface temperature (SST) on its margin during Heinrich stadial (HS) 4 and Greenland interstadial (GI) 8. **(A)** Air and sea surface winter temperatures during HS 4, **(B)** air and sea surface winter temperature during GI 8, **(C)** annual precipitation during HS 4, and **(D)** annual precipitation during GI 8. These maps interpolate the temperatures and precipitation estimated from four cores strategically located in the Atlantic and Mediterranean regions (Sánchez Goñi et al., 2002 and unpublished data). Black circles indicate the location of cores MD95-2043, MD95-2042, MD99-2331, and MD04-2845 (Naughton et al., 2007; Fletcher and Sánchez Goñi, 2008; Sánchez Goñi et al., 2008).

The concentration (usually between 1,000 and 50,000 grains cm^-3^) and taxonomical diversity of marine pollen assemblages are in general high enough to allow the quantitative estimation of annual and, more importantly, seasonal temperatures and precipitation (e.g., Sánchez Goñi et al., 2002, 2005, 2016a; Desprat et al., 2007; Combourieu Nebout et al., 2009) (**Figure 3**). Knowledge about past climate seasonality is crucial to understand natural climate variability (Carré and Cheddadi, 2017) and the sensitivity of vegetation to changes in this parameter makes pollen data particularly well-suited to track shifts in seasonality through time. Quantitative pollen-based climatic estimates are based on different techniques. The most frequently used is the Modern Analog Technique (MAT) based on a comparison of past assemblages to modern pollen assemblages through the calculation of the shortest weighted distance (Guiot, 1990). This method requires high-quality, taxonomically consistent modern pollen and climate datasets. The modern database includes different continental pollen spectra (moss polsters, soil surface samples, and lacustrine sediments) from Eurasia and northern Africa (Peyron et al., 1998; Bordon et al., 2009). This continental pollen database is, however, slightly modified to estimate climatic parameters from marine pollen samples. Since *Pinus* pollen is overrepresented in marine sediments (Heusser and Balsam, 1977; Hooghiemstra et al., 1988), this pollen type is excluded from the calculation of pollen percentages for marine pollen spectra as well as from the continental pollen database. As potential errors would be similar for adjacent samples of the same record, we consider that this approach is still valid for the quantification of climate change anomalies. The application of the MAT to estimate quantitatively climatic parameters for marine pollen records should be further improved by integrating marine modern pollen assemblages in the database, and by application of other reconstruction approaches that rely less strongly on the availability of suitable modern analogs, such as indicator approaches (Kühl et al., 2002) and biomisation (Prentice et al., 1996).

### A Global Compilation of Deep-Sea Pollen Records

**FIGURE 4:**
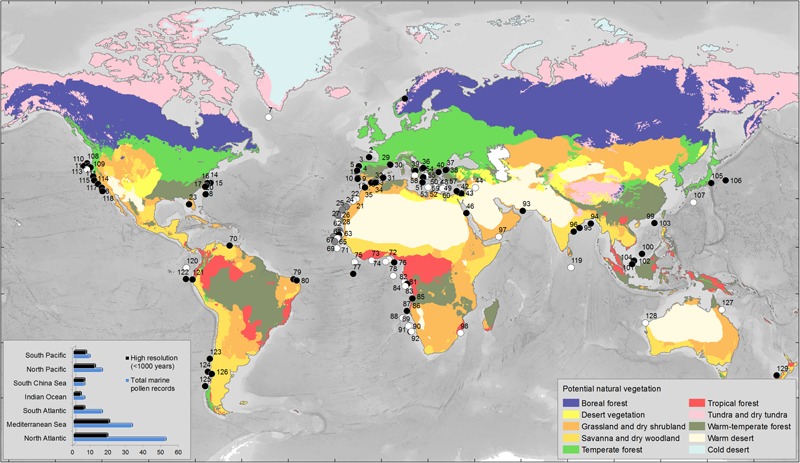
Global map with the main types of potential vegetation (Levavasseur et al., 2012) and the marine pollen records covering different intervals of the last 2.58 Myrs. We have restricted the selection to pollen records from sections cored in the present-day marine realm. Black circles indicate the pollen records with a temporal resolution better than 1000 years (<1000 years) between adjacent samples. White circles indicate the pollen records with a temporal resolution worse than 1000 years (>1000 years) between adjacent samples. Gray circles indicate records where no information about the resolution is given. Left bottom corner: histogram indicating the number of marine pollen records by region and the number of high resolution records among the 129 records compiled in this work (see **Supplementary Table S1** for details and references). Warm-temperate forest biome includes the Mediteranean forest; Temperate forest biome includes the Atlantic forest; Grassland and dry shrubland include the Mediterranean plants, i.e., sclerophyllous trees and shrubs such as *Quercus ilex* type (holm oak, kermes oak), *Q. suber* type (cork oak), *Olea* (olive tree), *Phillyrea, Pistacia*, and *Cistus* (rockroses); Desert and Tundra-dry tundra are the present-day closest vegetation biomes to the past semi-desert and steppe environments inferred from the pollen analysis of last glacial sedimentary sequences, respectively.

During the last 60 years, several oceanographic cruises organized in the framework of the international programs IMAGES (International Marine Global changE Study) and ODP/IODP (International Ocean Drilling Program) have retrieved a large number of pollen-rich marine cores along different oceanic margins. Most of these cores come from oceanic rises and abyssal plains that are the most favorable areas for marine palynological work as terrigenous sediments are predominant (Groot and Groot, 1966). We have performed a literature survey to compile pollen records from deep-sea cores spanning the last 1 million years, finding 129 sites all over the world (**Supplementary Table S1** and **Figure 4**). This compilation shows that there are 74 high-resolution pollen sequences (better than 1000 years between adjacent samples) and highlights the paucity of pollen records from the Indian (e.g., Rossignol-Strick, 1983) and the South Pacific (e.g., Montade et al., 2013; Seillès et al., 2016) Oceans. Likewise, almost half of the records (*n* = 57) do not cover periods older than the Last Glacial Maximum, and few sequences (*n* = 19) record several orbital climatic cycles. Therefore, the regional expression of long-term and rapid, millennial-scale, global climate changes is far from being well-documented, particularly in the Indian Ocean where only two out of the six available records cover the last climatic cycle (last 150,000 years) with a coarse time resolution (worse than 1000 years between consecutive samples). To fill this gap, IODP expedition 353 “Indian Monsoon Rainfall” collected a sedimentary sequence in 2015 on the eastern Indian margin, site U1446, which will enable to trace for the first time past vegetation and Indian summer monsoon variability over the last 1 million years.

## European Vegetation and Climate Response to Long-Term and Rapid Climate Variability

### General Setting

Since 1995, the Marion Dufresne and Joides Resolution oceanographic ships have retrieved several high-resolution pollen-rich deep-sea sedimentary sequences from the eastern North Atlantic and the Mediterranean Sea within the framework of the IMAGES and IODP programs. These sequences are strategically located in the present-day Mediterranean and Atlantic regions, characterized by a markedly seasonal climate, with warm and dry summers and wet and mild winters, and a year-round humid climate with warm summers and cool winters, respectively. In winter, both regions are under the influence of the westerlies, but in summer the Azores high affects southern Iberia inducing a pronounced summer drought while the westerlies still play a prominent role from northern Iberia northward. Additionally, the region located below 40°N is affected by precession, and the subtropical ocean water currents (Ruddiman and Mcintyre, 1984).

At present, the seasonal distribution of precipitation, with a relatively abundant amount of rainfall from autumn to spring and a pronounced summer drought, determines the dominance of Mediterranean forest (Polunin and Walters, 1985; Gouveia et al., 2008). This forest comprises evergreen sclerophyllous woodland (evergreen *Quercus, Olea, Pistacia, Phillyrea, Cistus*) at low altitudes, and deciduous forest (mostly deciduous *Quercus*) at higher elevation. On the contrary, the occurrence of Atlantic forest, dominated by deciduous *Quercus* (oak), is today controlled by winter length and harshness, given that annual rainfall is high and summer drought absent or negligible (Polunin and Walters, 1985). Therefore, deciduous *Quercus* is included in both the ‘Atlantic forest’ and the ‘Mediterranean forest’ assemblages.

Pollen analysis of these European margin sequences has allowed the reconstruction of the responses of western European vegetation and climate to long- and short-term global climate changes of the Ice Ages.

### European Vegetation and Climate Response to Past Global Long-term Climate Changes

Geological archives show that the Earth’s climate has experienced large changes during the last 2.58 million years (Shackleton and Opdyke, 1973; Hays et al., 1976), with alternating warm (interglacial) and cold (glacial) periods. During the glacial periods ice occupied large regions of the Northern Hemisphere resulting in low sea level stands while during interglacials the ice sheets only covered Greenland and sea level was similar or higher than at present. These changes in ice volume are identified in marine sedimentary sequences by measuring the oxygen isotope composition (δ^18^O) of benthic foraminifer carbonate shells, providing the basis of the marine isotopic stratigraphy. Depleted values define the interglacial periods *sensu lato* (s.l.) or odd numbered MIS, while heavy values mark the glacial periods corresponding to even-numbered MIS. Within interglacial periods, an alternation of ice retreats, called “e,” “c” and “a,” and ice advances, “d” and “b,” is observed, also resulting from orbital forcing. The interglacial period *sensu stricto* (s.s.) corresponds to the substage with minimal ice volume, often located just after deglaciation (Railsback et al., 2015).

**FIGURE 5:**
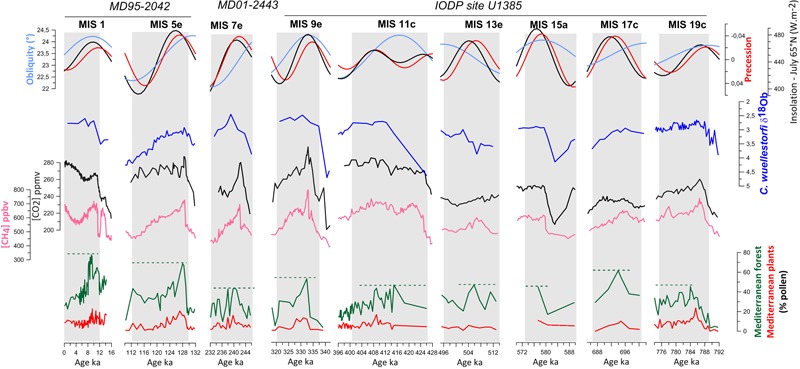
Pollen and isotopic (δ^18^O_b_) records from the southwestern Iberian margin (Sánchez Goñi et al., 1999, 2016b; Roucoux et al., 2006; Chabaud et al., 2014; Hodell et al., 2015; Oliveira et al., 2016; and unpublished data). The Mediterranean forest is mainly composed of deciduous oaks and Mediterranean plants, i.e., sclerophyllous trees and shrubs such as *Quercus ilex* type (holm oak, kermes oak), *Q. suber* type (cork oak), *Olea* (olive tree), *Phillyrea, Pistacia*, and *Cistus* (rockroses). Pollen data are compared to CO_2_ and CH_4_ records from ice cores (Siegenthaler et al., 2005; Spahni et al., 2005; Loulergue et al., 2008; Luthi et al., 2008) and astronomical forcing (Berger, 1978). Adapted from Desprat et al. (2017).

Two long and continuous pollen-rich sedimentary sequences are available from the southwestern and northwestern Iberian margin covering the last 1.5 million years and 425,000 years, respectively. These Iberian margin sequences are each composed of three cores: the southern sequence is a composite of cores MD95-2042, MD01-2443, and IODP U1385 located at around 37°N (**Figure 5**; Sánchez Goñi et al., 1999, 2016b; Roucoux et al., 2006; Chabaud et al., 2014; Oliveira et al., 2016), and the northern one is a composite of cores MD99-2331, MD01-2447, and MD03-2697 collected at 42°N (Desprat et al., 2005, 2006, 2007, 2009; Sánchez Goñi et al., 2005, 2008; Naughton et al., 2007, 2009, 2016). The pollen analysis of the 425,000-year northern sequence is completed (Desprat et al., 2017) while that of the southern site is in progress (**Figure 5**). Further north, the sequence MD04-2845 collected on the European margin, off western France, spans the last 140,000 years (Sánchez Goñi et al., 2008, 2012).

Both Iberian margin pollen sequences show an alternation of glacial periods dominated by Mediterranean steppe in southern Iberia (that we call semi-desert although it rarely included true semi-desert taxa such as *Lygeum*) and steppe/heathlands to the north, and interglacial periods when Mediterranean and Atlantic forests expanded over southern and northern Iberia, respectively. Tree pollen percentages are a good estimate for the magnitude of the interglacial in both Iberian regions. The magnitude of past Mediterranean forest expansions is interpreted as an indicator of the amount of winter precipitation in southern Iberia, given that relatively warm temperatures and summer drought occurred. The magnitude of the Atlantic forest expansion during different interglacials will mainly reflect the amplitude of the warming above 40°N, as forest development in the Atlantic domain requires a climate with a growth period of 4–6 months, and a mild winter period of 3–4 months (Polunin and Walters, 1985). Two or even three major phases of forest expansion occurred during all these interglacials, related with ice volume minima during sub-stages “a,” “c,” and “e” within each MIS (**Figure 5**). However, forest development in general reached its maximum during the earliest phase of each stage, concomitant with the highest sea level, indicating that warmest and wettest conditions occurred then, and subsequently identifying this phase with the terrestrial interglacial *sensu stricto*.

Pollen records from the Iberian margin show that the magnitude of forest development substantially differed from one interglacial to another in the south (MIS 19 to MIS 1, Mediterranean forest pollen = 40 to 80%, **Figure 5**), while in the north the magnitude of the forest expansion was similar (Atlantic forest pollen = 80%; Desprat et al., 2017). These data suggest that temperatures were similarly warm during the last five interglacials in northwestern Iberia, whereas the amount of winter precipitation in the south was quite variable. The Holocene (MIS 1, ∼10 ka), the last interglacial (MIS 5e, ∼128 ka) and MIS 17c (∼690 ka) would have been the wettest followed by MIS 9e (∼335 ka), MIS 11c (∼415 ka) and MIS 19c (∼784 ka), and MIS 7e (∼240 ka) being the driest. Excluding the Lake Orhid pollen record (Sadori et al., 2016), these results coincide with what is observed in the European sequences of Tenaghi Philippon and Praclaux located above 40°N (Reille et al., 2000; de Beaulieu et al., 2001; Tzedakis et al., 2006), which show similar expansions of the temperate forest during the interglacials of the last 400,000 years. So far no long terrestrial pollen sequence exists below 40°N for comparison with the southwestern Iberian margin pollen record.

The comparison of the Iberian observations with those at higher latitudes illustrates the regional variability of the magnitude and impacts of climate change. Due to the combined forcing of insolation and GHG, climatic models indicate that MIS 5e, MIS 9e, and MIS 11c were the warmest at the highest latitudes, associated with the strongest melting of ice sheets (Yin and Berger, 2012, 2015). This scenario is in marked contrast with data from southwestern Europe showing that these interglacials were not particularly warm. Our pollen data suggest that substantial differences in precipitation occurred across the different interglacials. Nevertheless, it is difficult to propose a relationship between warming at high latitudes due to GHG increase and dryness in the Mediterranean region, contrary to what climate models project for the end of this century (Hoerling et al., 2012). Identifying forcing factors responsible for the interglacial diversity in terms of precipitation in the Mediterranean region is a subject of debate. A good candidate is the orbital parameter of precession that influences regions below 40°N (Ruddiman and Mcintyre, 1984). Precession controls the amplitude of the seasonality and, therefore, the amount of winter precipitation in subtropical regions as recently suggested by models (Yin and Berger, 2012) and observations (e.g., Sánchez Goñi et al., 2008; Oliveira et al., 2016, 2017).

### Rapid Climate Changes: A Focus on the Last Glacial Period

More recently, geological archives have also shown that millennial-scale changes punctuated this long-term climatic variability at the global scale as these changes are registered in North Atlantic SSTs (Voelker and Workshop Participant, 2002; Martrat et al., 2007) as well as in air temperatures over Greenland (North Greenland Ice-Core Project Members [NGRIP], 2004; Barker et al., 2011) and Antarctica (Augustin et al., 2004). These changes occur independently of the boundary climate state, i.e., in glacial and interglacial periods, and are generally associated with changes in ice volume (Siddall et al., 2003), GHG concentration (Knutti et al., 2004; Spahni et al., 2005; Loulergue et al., 2008), and AMOC (Oppo et al., 2006; Lynch-Stieglitz, 2017). The magnitude and frequency of these short-term climatic oscillations were larger when ice caps reached a critical mass (McManus et al., 1999) even if they were not the trigger (Barker et al., 2015).

So far, the best studied millennial-scale climate variability is that observed during the last glacial period (MIS 4, MIS 3 and MIS 2, i.e., ∼73–14.7 ka) characterized by a series of warming and cooling events called Dansgaard-Oeschger (D-O) cycles that were first identified in the atmosphere of Greenland and usually lasted 500–2,000 years (Dansgaard et al., 1984). These cycles recorded in the δ^18^O of the ice cores were characterized by a large (7 to 16°C) and rapid (within a few decades) warming event followed by a progressive decrease in temperature and a final abrupt cooling (Wolff et al., 2010). The warming and progressive cooling phase is termed Greenland Interstadial (GI), and the final cooling leading to the cold phase termed Greenland Stadial (GS). The GI phases lasted between 100 and 2,600 years (Wolff et al., 2010). Also during the last glacial period, repeated iceberg discharges cooled the surface of the North Atlantic (Heinrich, 1988; Bond et al., 1993). On the one hand, massive iceberg discharges from the Laurentide ice sheet, the so-called Heinrich events (HE), occurred with a cyclicity of 7,000–10,000 years while, on the other hand, weaker discharges coincided with iceberg fragmentation from the British-Icelandic-Scandinavian (BIS) ice cap (Elliot et al., 2001). We define a HE as the time period synchronous with the deposition of the coarse sediment Heinrich layer in a given region following the iceberg discharge while the Heinrich Stadial (HS) is the cold interval associated with the HE (Sánchez Goñi and Harrison, 2010). Some GSs encompass the HSs while the others are associated with the BIS minor iceberg discharges. These cold intervals are related to decreases in the AMOC (McManus et al., 2004; Lynch-Stieglitz, 2017) but the cause of the ice sheet collapse remains a subject of debate (e.g., Alvarez-Solas and Ramstein, 2011; Barker et al., 2015). The duration of the iceberg discharge has been simulated to be between 50 and 200 years (Roche et al., 2004) and data show that the HSs lasted longer, up to 3000 years (Sánchez Goñi and Harrison, 2010). HE and HS time intervals coincide in the Ruddiman belt, the preferential zone of IRD deposition that extends roughly between 45 and 50°N, while this is not the case outside (Naughton et al., 2009). Since the discovery of these millennial-scale climate changes, D-O cycles and HEs, the paleoclimatic community has dedicated major efforts to investigate the regional expression of this variability, the oceanic and atmospheric mechanisms involved in its transmission, and the interaction with long-term climate forcing such as the orbital parameters and ice volume. The following sub-section summarizes the great contribution that palynological research conducted on marine sediments has done so far to increase current understanding of these processes in western Europe.

#### D-O Cycles

**FIGURE 6:**
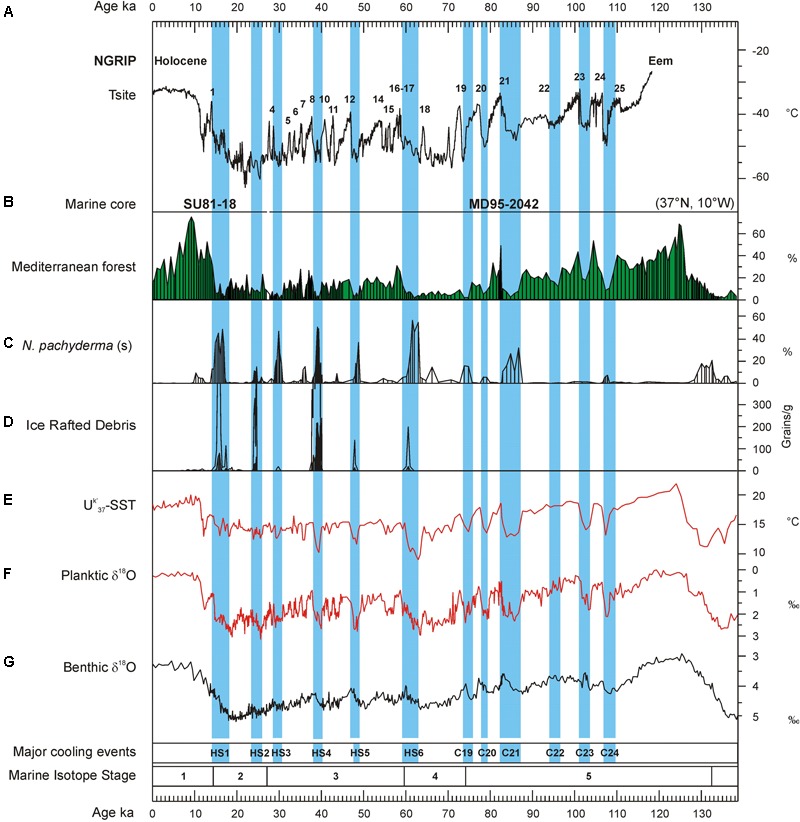
Vegetation response to the millennial-scale variability of the last glacial period in southwestern Iberia. Terrestrial and marine palaeoclimatic record from the twin cores MD95-2042 and SU81-18 (SW Iberian margin) for the last 138 ka compared with Greenland temperature variations. **(A)** Greenland temperature curve (100 years resolution) derived from air and water isotopic measurements. Numbers 1–25 refer to the D–O warming events, **(B)** pollen percentage curve of the Mediterranean forest (deciduous and evergreen *Quercus, Olea, Phillyrea, Pistacia*, and *Cistus*), **(C)** percentage curve of the polar planktonic foraminifer *Neogloboquadrina pachyderma* (s), **(D)** concentration curve of the ice-rafted debris (IRD, in grains per gram of dry sediment) reflecting the main episodes of iceberg melting in the Iberian margin. Thin line indicates exaggeration x10, **(E)** curve of the alkenone-derived (Uk′_37_) sea-surface temperatures (SST), **(F)** planktic foraminiferl δ^18^O curve on Globigerina bulloides, **(G)** benthic foraminifer δ^18^O curve indicating the marine isotopic stratigraphy. Blue intervals indicate the Heinrich stadials associated with ice-rafting events of the last climatic cycle: HS1–HS6 and C19–C24 (Bond et al., 1993; Chapman and Shackleton, 1999). N.B.: for the last 25,000 years, the IRD, *N. pachyderma* (s) and pollen data are from the twin core SU81-18 (Turon et al., 2003) that are similar to that obtained from core MD95-2942 (Chabaud et al., 2014). Adapted from Sánchez Goñi et al. (2008).

Until the end of the 1990s, investigations into European pollen records reported no changes in the dominant steppe vegetation through the last glacial period (e.g., Follieri et al., 1988; Pons and Reille, 1988; Reille and de Beaulieu, 1990). Allen et al. (1999) published a pollen record from southern Italy showing for the first time millennial-scale changes between forest expansion and contraction that corresponded to the rapid climate variability detected in Greenland and the North Atlantic Ocean. However, the chronological uncertainties between ice, terrestrial and marine sequences precluded the demonstration of whether forest expansions corresponded to North Atlantic and Greenland warming or, inversely, to cooling events. Marine pollen records have unequivocally shown that cooling events in the North Atlantic and high latitudes corresponded in western Europe with herbaceous community expansion and warming events with enhanced forest development (Roucoux, 2000; Sánchez Goñi et al., 2000, 2002, 2008). The aforementioned marine pollen records from the European margin and an additional record, MD95-2043, from the Alboran Sea (western Mediterranean, **Figure 4**) (Sánchez Goñi et al., 2002; Fletcher and Sánchez Goñi, 2008) also show that vegetation quickly, within 100 years, responded to the D-O cycles and HEs, and that there was a dynamical equilibrium between vegetation response and climate change for short periods of forcing. Cold episodes in North Atlantic surface temperatures related to GS, not only those associated with HEs, were synchronous with forest reductions in Iberia and western France, while forest expansions correlated with increases in SST (**Figure 6**) (Sánchez Goñi et al., 2008).

**FIGURE 7:**
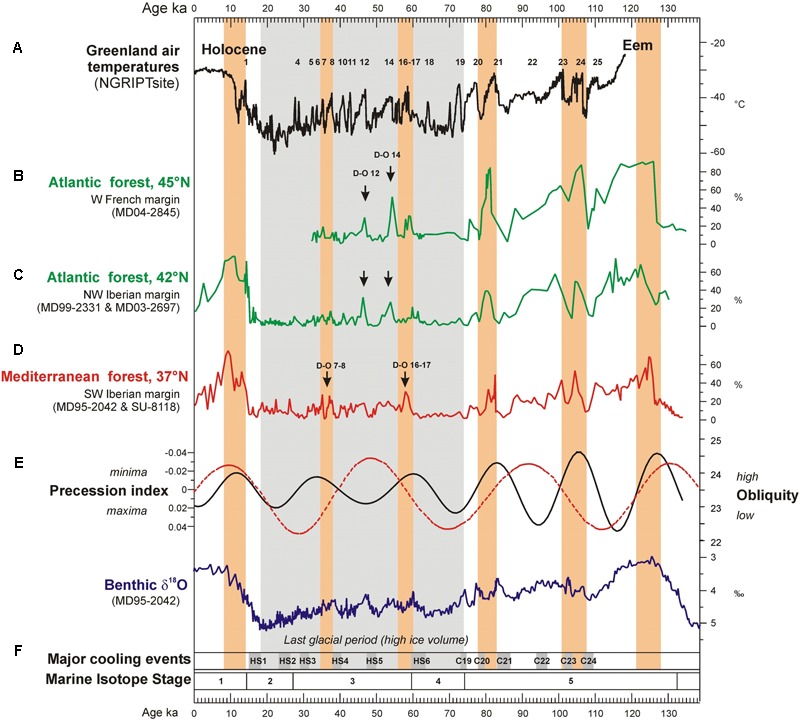
Evolution of Greenland temperature, western European temperate forest development (37–45°N) and SSTs (37°N) over the last climatic cycle compared with iceberg discharges (37°N), precession index, obliquity and ice volume variations. **(A)** Greenland temperature reconstruction (100 years resolution). Numbers 1 to 24 indicates D-O warming events, **(B)** Atlantic forest pollen percentages in western France from deep-sea core MD04-2845 (136–35 ka), **(C)** Atlantic forest pollen percentages in northwestern Iberia from deep-sea cores MD99-2331 (131–18 ka) and MD03-2697 (14–0 ka), **(D)** Mediterranean forest pollen percentages in southwestern Iberia from deep-sea cores MD95-2042 (27–134 ka) and SU8118 (0–27 ka), **(E)** precession index and obliquity, **(F)** benthic δ18O curve from deep-sea core MD95-2042. Gray rectangles indicate the North Atlantic ice-rafting events of the last climatic cycle: HS1 to HS6 and C19 to C24 (Bond and Lotti, 1995; Chapman and Shackleton, 1999). Orange rectangles indicate the maximum expansion of the Mediterranean forest. Adapted from Sánchez Goñi et al. (2008).

Notably, below 40°N, Mediterranean forest reached its maximum development at the onset of the Eemian (MIS 5), the D-O 24, D-O 21, D-O 17-16, D-O 8-7, and D-O 1 warming events, and the beginning of the Holocene (MIS 1), indicating the occurrence of enhanced hot/dry summers and wet/cool winters (**Figure 7**). The Atlantic sites, above 40°N, showed a contrasting pattern: during GI 12 and GI 14 the Atlantic forest experienced a strong development while the impact of D-O 17-16 and D-O 8-7 warming was rather limited. The comparison of the Mediterranean and Atlantic palaeoclimatic records reveal that there was a spatial variability in the amplitude of the forest expansions for any given D-O warming of the last glacial period (Sánchez Goñi et al., 2008) (**Figure 7**). The comparison of these changes in vegetation with the evolution of orbital parameters shows that the maxima in the Mediterranean forest were always synchronous with low precession values. This observation suggests that precession minima strengthened Mediterranean climate through promoting marked seasonality. The floristic composition of the Mediterranean forest corroborates that Mediterranean climate reached its best expression during precession minima (Sánchez Goñi et al., 2008). For example, pollen data from the Alboran Sea core show that Mediterranean sclerophylls such as evergreen *Quercus, Olea, Cistus, Phillyrea, Coriaria myrtifolia*, and *Pistacia* were particularly abundant during GI 8, under enhanced seasonality associated to a minimum in precession (Fletcher and Sánchez Goñi, 2008). On the contrary, the same sequence records that the abundance of the less drought-tolerant Ericaceae (heather) was higher during GI 12, when precession reached a maximum and seasonality was reduced (Fletcher and Sánchez Goñi, 2008). In contrast, the maximum expansion of the Atlantic forest coincides with maxima in obliquity. For northern latitudes above 40°N obliquity seems to play a major role in modulating the increase of annual temperatures (**Figure 7**). The amplitude of temperature changes in Greenland was strong at D-O 17-16, D-O 12 and D-O 8, and weak at D-O 14 (Sánchez Goñi et al., 2008), thus also differing from the climatic patterns at lower latitudes. This means that the strongest warming events in Greenland were not necessarily particularly warm in other regions of the world. The Iberian Peninsula and wider Mediterranean region are particularly interesting from a palaeoclimatic point of view because they appear to straddle the transition zone from obliquity- to precession-influence on the climatic signal of millennial-scale warming events. Preliminary comparison between European margin and terrestrial pollen records across Europe roughly confirms the contrasting latitudinal response over Europe to the D-O warming events despite the independent and sometimes uncertain chronologies of individual records (Fletcher et al., 2010). A more in-depth comparison of these pollen records is in progress based on the harmonized chronology recently developed in the framework of the INQUA International Focus Group (IFG) ACER project (Sánchez Goñi et al., 2017).

**FIGURE 8:**
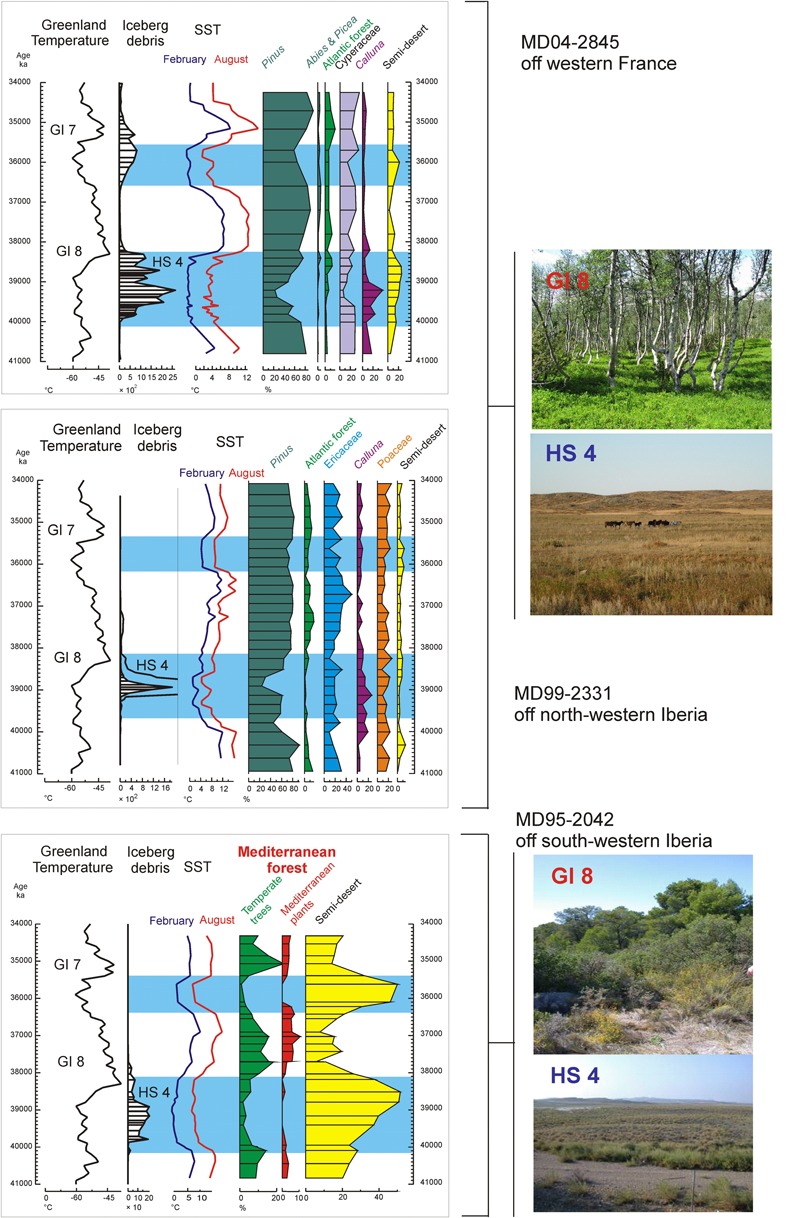
A “zoom” on the interval 41–34 ka, from the three western European margin cores. The location of these cores is shown in **Figure 3**. GI (Greenland Interstadial), HS (Heinrich Stadial), GS (Greenland Stadial). In contrast with the Atlantic region, *Pinus* pollen percentages in core MD95-2042 (not shown) are similar through all the considered interval, during GI and GS, and oscillate between 60 and 90%. The pictures named HS 4 and GI 8 suggest analog present-day landscapes: semi-desert (*Ephedra distachya*-type, *E. fragilis*-type, Chenopodiaceae that now is included in the Amaranthaceae pollen morphotype, *Artemisia*) and open forest in the Mediterranean region and steppe (Poaceae, Cyperaceae, Asteraceae, Ericaceae and semi-desert plants) and *Pinus–Betula* forest in central Europe, respectively. SST: sea surface temperatures estimate from planktonic foraminifer assemblages using the Modern Analog Technique (MAT) (Guiot and De Vernal, 2007). Blue rectangles indicate the cold intervals, HS 4 and GS 8. Adapted from Sánchez Goñi (2014).

A zoom into D-O 7 and D-O 8 cycles, 41,000–34,000 ka, shows that semi-desert plants (mainly *Artemisia*, Chenopodiaceae and *Ephedra*) characterized the vegetation of southern Iberia during GSs, with open Mediterranean woodland establishing during GIs (Sánchez Goñi et al., 2008; **Figure 8**). Simultaneously, Ericaceae and Poaceae dominated the vegetation of north-western Iberia during GSs (**Figure 8**), being partially replaced by Atlantic woodlands with *Pinus* and deciduous *Quercus* during warmer periods (GIs). The composition of the pinewoods of south-western/central Iberia during the last glacial also varied in response to abrupt climate changes according to the quantitative study of *Pinus* pollen grains by Desprat et al. (2015). Thus, although *P. nigra* was the dominant pine species throughout the last glacial period, *P. sylvestris* was more abundant during the colder GSs and HSs and Mediterranean pines (*P. pinaster, P. pinea, P. halepensis*) during the warmer GIs and early- to mid-Holocene. Further north, *Artemisia*, Cyperaceae and *Calluna* dominated in western France during the cold periods, whereas *Betula*, deciduous *Quercus* and conifers (*Pinus, Abies, Picea*) expanded slightly in the landscape during warming episodes (Sánchez Goñi et al., 2008; **Figure 8**). These sequences illustrate the spatial floristic diversity of western Europe in response to the D-O cycles. Interestingly, the latitudinal boundary between the Atlantic and the Mediterranean vegetation during the last glacial period seems to have been similar to that at present-day, i.e., below 40°N. Finally, it is worth highlighting that temperate forest expanded when summer SST in the North Atlantic crossed the threshold of 12°C (Sánchez Goñi et al., 2008) (**Figure 8**), which is the same threshold value that explains the distribution of the temperate forest in both sides of the North Atlantic Ocean at present (Van Campo, 1984).

#### A Close-up on HS and GI Phases

**FIGURE 9:**
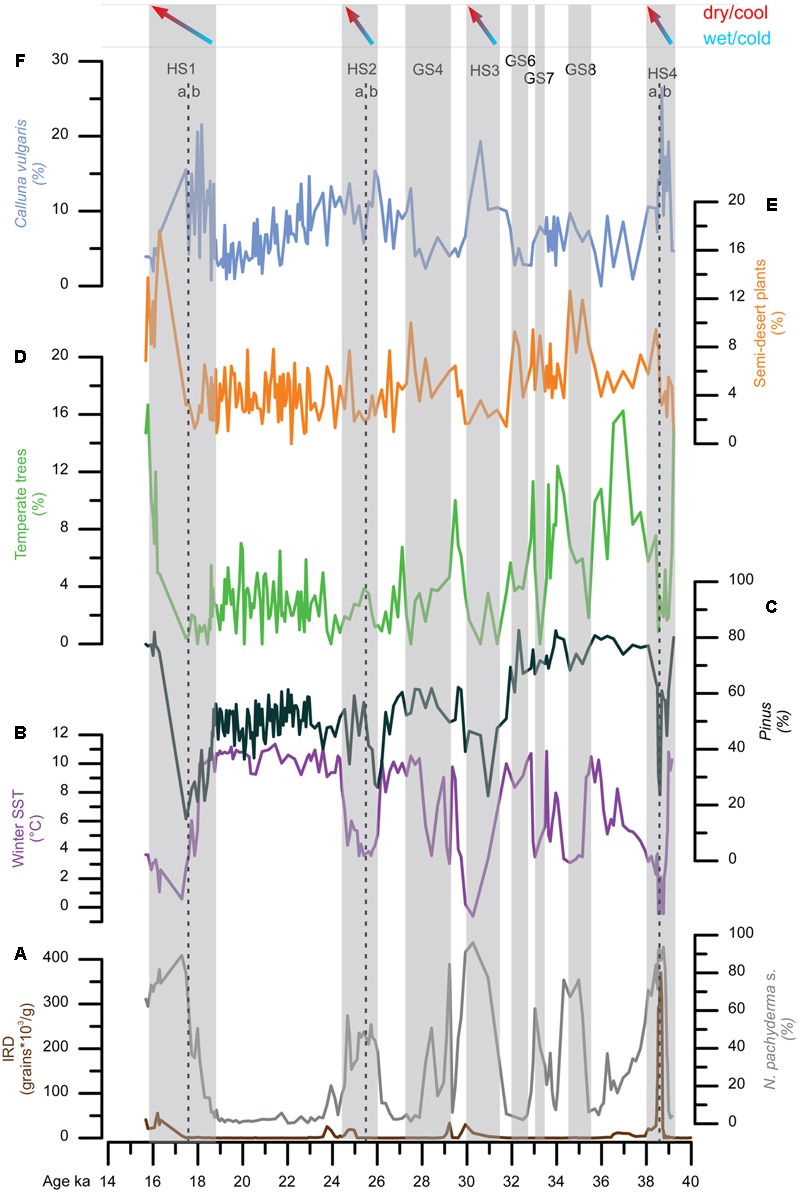
Direct land-sea correlation of MD99-2331 record. From bottom to top: **(A)** ice-rafted debris (IRD) concentrations (brown line), percentages of polar planktonic foraminifer [*N. pachyderma* (s)] (gray line), **(B)** winter SST estimates based on planktonic foraminifer associations, **(C)** pollen percentages of temperate trees (*Acer, Alnus, Betula, Corylus, Juniperus-Cupressus*-type, deciduous and evergreen *Quercus, Fraxinus excelsior*-type, *Salix, Tilia*, and *Ulmus*), and *Pinus*, **(D)** pollen percentages of **(E)** semi-desert plants and **(F)** *Calluna vulgaris*. Gray bars represent the HS 4, HS 3 (∼GS 5), HS 2 (∼GS 3) and HS 1 and GS 8, GS 7, GS 6, GS 4. HS 4, HS 3 and HS 1 are divided in two phases, a and b. HS 4 (a: ∼39.5–38.6 ka; b: ∼38.6–38 ka); HS 2 (a: ∼26–25.5 ka; b: ∼25.5–24 ka); HS 1 (a: ∼18.8–17.5 ka; b: ∼17.5–15.8 ka). Adapted from Naughton et al. (2009).

Marine pollen records unequivocally show that HSs were associated with the expansion of herbaceous vegetation reflecting cold and dry conditions in western Europe. However, a closer observation of HS 4 (40.2–38.3 ka), HS 2 (26.5–24.3 ka), and HS 1 (18–15.6 ka) in core MD99-2331 retrieved off northwestern Iberia suggests that two major phases occurred within these stadials (**Figure 9**) (Naughton et al., 2009). The first phase was characterized by almost no IRD in the north-western Iberian margin but SSTs were low and large reductions of *Pinus* and Atlantic forest pollen indicate very cold atmospheric conditions as well. The first episode was the wettest one according to the maximum percentages of *Calluna*, typical at present of central European mires (Polunin and Walters, 1985), and high pollen concentration (not shown). The second phase of these stadials was drier and warmer as shown by the increase of semi-desert plants, and *Pinus* and temperate forest pollen percentages, respectively.

**FIGURE 10:**
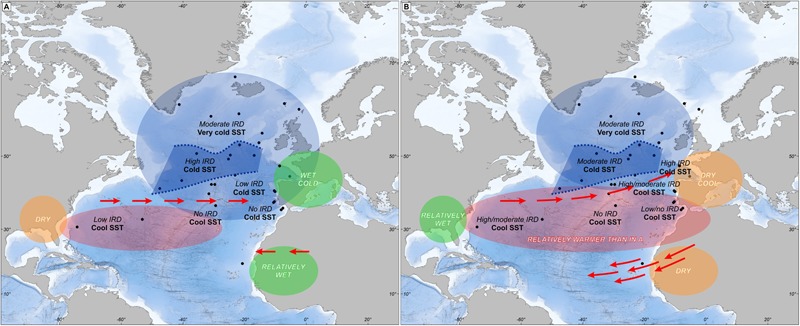
Mapping oceanic and atmospheric patterns of Heinrich events in the North Atlantic region within: **(A)** the first phase and **(B)** the second phase of HS 4, HS 2 and HS 1. The gray zone in the middle of the North Atlantic represents the Ruddiman belt. Adapted from Naughton et al. (2009).

A synthesis of all available North Atlantic sites recording the last four HS shows complex climatic and IRD deposition patterns that are summarized in **Figure 10** (Naughton et al., 2009). In the first phase, the wettest and coldest conditions in the Iberian Peninsula were coetaneous with low IRD deposition and low SST in the Iberian margin. A contrasting pattern is detected in the Ruddiman belt (45–50°N) with high IRD deposition and cold SST while in Florida a dry climate was associated with cool SST off this peninsula. During the second phase, dryness in Iberia and high IRD deposition in its margin are observed at the same time as wetness in Florida and moderate IRD deposition in the Ruddiman belt. A simple oceanographic mechanism related to changes in the strength of the AMOC alone cannot explain this complex scenario but rather an atmospheric mechanism must be involved. During the first phase, strong SST cooling of the North Atlantic as far as the southern Azores and the Gulf of Cádiz was associated with relatively high precipitation in south-western Europe. At the same time, δ^13^C measurements on benthic foraminifer and Pa/Th ratios indicate a gradual decrease of deep sea ventilation and a slowdown of the AMOC, respectively (Naughton et al., 2009). The southward displacement of the thermal front produced a strong mid-latitude meridional temperature gradient that led to the southward displacement of the westerlies and increased precipitation in southwestern Europe. The scenario highlighted by our synthesis suggests that a mechanism similar to that of the present-day NAO in negative mode could have played a role (Naughton et al., 2009).

During the second phase of HS4, HS2 and HS1, climatic proxies indicate a northward migration of the thermal front associated with the northward shift of the westerlies and dryness in western Europe. This is accompanied in simulations by the southward displacement of the Inter Tropical Convergence Zone (ITCZ) (Kageyama et al., 2009) as detected from the increased of trade winds offshore Senegal (Jullien et al., 2007). As a whole, this configuration is similar to that currently produced by the positive mode of the NAO (Naughton et al., 2009). A marine pollen record from the Bay of Bengal indicates that HS 2 was associated with extremely weak summer and winter Indian monsoons, thus adding support for the southward migration of the ITCZ (Zorzi et al., 2015) during this period as also shown by South American and Asian speleothem records (e.g., Wang et al., 2004, 2008) and other marine sequences off western Africa (e.g., Weldeab et al., 2007).

The multi-phase structure of HS 4, HS 2 and HS 1 is supported by changes in ^17^O-excess, increase in CO_2_ and methane mixing ratio and heavier δD-CH_4_ and δ^18^O_atm_ observed in Greenland ice cores (Guillevic et al., 2014). In particular, in the second part of GS 9 (39.9–38.1 ka) the HS 4 imprint in Greenland would be characterized by a lower-latitude signal (without no changes in high-latitude temperature) associated with the southward migration of the ITCZ and a cold Europe (Guillevic et al., 2014). These data evidence a decoupling during GS 9 between stable cold Greenland temperature and low-latitude climate variability.

A decoupling between high and lower latitudes is also observed during GI 12 (46.8–44.3 ka) and GI 8 (38.2–36.6 ka) (Sánchez Goñi et al., 2009). After the northern hemispheric rapid warming at the GS-GI transition, the trend during the first part of the GI is a Greenland cooling and an Iberian warming. This increase of the North Atlantic climatic gradient led to moisture transportation to Greenland from mid latitudes (lightest d-excess) and to a drying episode in Iberia. The subsequent temperature decrease in Greenland and Iberia associated with the precipitation increase in the latter region occurred when the major source of Greenland precipitation shifted to lower latitudes (d-excess increase). These examples of decoupling during GS and GI provide new targets for benchmarking climate model simulations and testing mechanisms associated with millennial variability (Guillevic et al., 2014).

## Interactions Between Millennial and Orbital Climate Variability: the Missing Piece of the Ice Age Puzzle?

Changes in insolation roughly explain the timing of deglaciation and glacial inception (Milankovitch, 1941; Shackleton and Opdyke, 1973; Hays et al., 1976). However, internal feedback processes are needed to explain the ice age cycles, and previous research has mostly focused on those loops related to atmospheric and oceanic circulation (e.g., Risebrobakken et al., 2007; Bar-Or et al., 2008), GHG concentrations (e.g., Ganopolski et al., 2016), and vegetation-albedo feedback (Crucifix and Loutre, 2002; Desprat et al., 2005; Sánchez Goñi et al., 2005). So far little attention has been paid to the interactions between shorter (millennial-scale) and longer (10,000 to 100,000 years) term climate variability that may amplify the original orbital forcing (Hodell, 2016). Another important missing piece of the Ice Age puzzle involves the still debated origin of the short-term climatic variability at high versus low latitudes, which has implications for the start of glaciations (Berger et al., 2006).

### Internal Mechanisms Linking Changes in Earth’s Orbit to Ice Ages

In this section, we firstly focus on the specific mechanisms linking changes in Earth’s orbit to Ice Ages taking as a test bed the onset of the last glaciation (i.e., MIS 5a/4 transition: ∼80–70 ka), when one of the largest ice accumulations of the last 250,000 years occurred.

Ruddiman and McIntyre (1979) showed unequivocally that the ice growth in the northern hemisphere was contemporaneous with persistent warmth and high salinity in the subpolar North Atlantic (44–54° N) when boreal summer insolation was decreasing. This apparent paradox was interpreted as the development of a strong thermal gradient between a warm subpolar North Atlantic and a cold nearby land leading to an increase of moisture. This moisture was transported toward the north by storm tracks and fell as snow following the decrease in insolation. However, this theoretical model involving a warm ocean-cold land thermal contrast was not confirmed at that time due to the lack of data. Also, the interglacial/glacial transitions were then thought to be gradual but we know now that they were punctuated by millennial-scale events. Thus, one may wonder whether sub-orbital climatic variability, i.e., D-O cycles and HEs, affected this orbitally-controlled ice growth.

**FIGURE 11:**
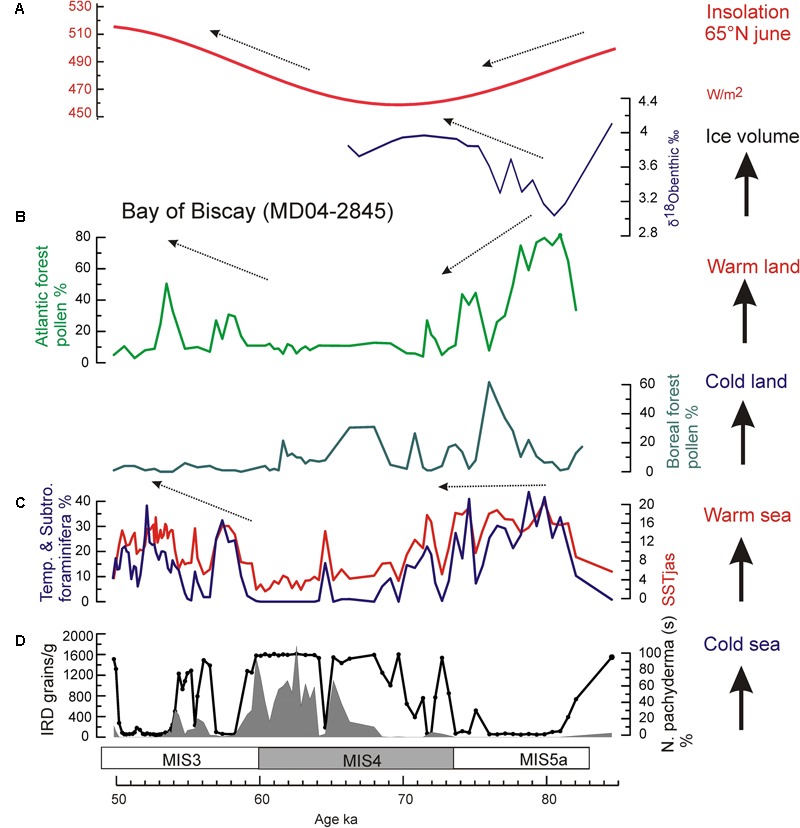
Insolation changes versus marine and terrestrial palaeoclimatic records from core MD04-2845 for the interval 85–50 ka (Sánchez Goñi et al., 2013): **(A)** insolation curve at 65°N in June (Berger, 1978), and δ^18^O benthic foraminifer isotopic record (*Cibicides wuellestorfi* > 150 μm), **(B)** pollen records of Atlantic and Boreal forests in western France, **(C)** foraminifer-based summer SST (red line) in the Bay of Biscay, and percentages of temperate and subtropical foraminifer assemblages (blue line), and **(D)** IRD concentration and *N. pachyderma* (s) percentage (black solid line) records.

**FIGURE 12:**
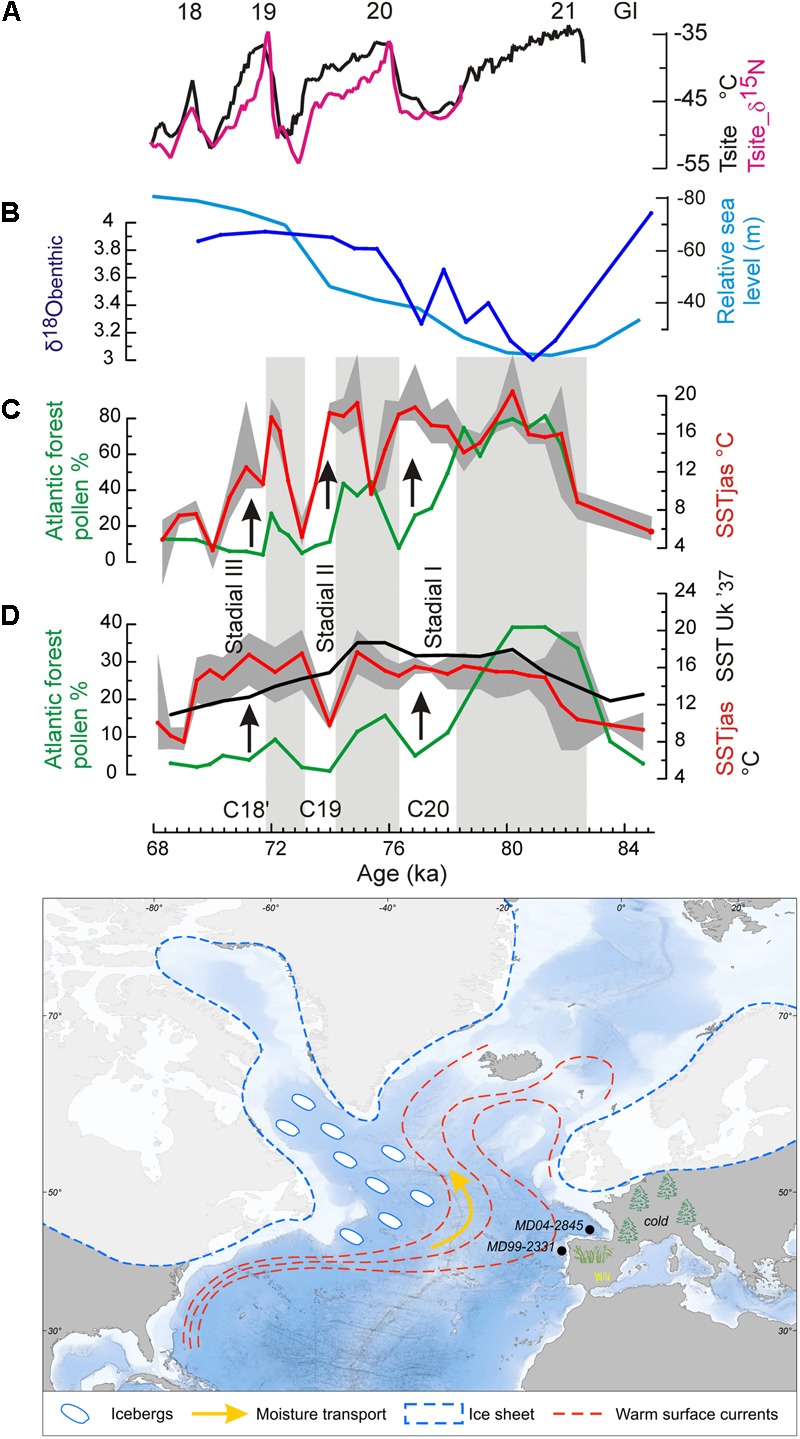
(Top) A “zoom” on the interval 84.2–68 ka. Greenland temperature record compared with land-sea palaeoclimatic records from the two western European margin cores during the MIS 5a/4 transition (Sánchez Goñi et al., 2013). **(A)** Greenland temperature record (Landais et al., 2004), **(B)** δ^18^O curve of benthic foraminifer from core MD04-2845 (dark blue), and reconstructed sea level changes (light blue) (Waelbroeck et al., 2002), **(C)** core MD04-2845 (Bay of Biscay): foraminifer-based summer SST curve (red) with the minima and maxima values found in the set of the five selected analogs (gray surface), Atlantic forest pollen percentage curves (green), **(D)** MD99-2331 (NW Iberian margin) the same as core MD04-2845, and Uk′37-based SST (black). Gray bands indicate warm intervals in western France. C20, C19, and C18′: cooling events in the western North Atlantic Ocean. GI 21, GI 20, GI 19, and GI 18: warm phases in Greenland. (Bottom) Schematic representation of the cold air-warm ocean contrast in the western European margin at the time of moderate iceberg discharges in the western North Atlantic Ocean.

During the MIS 5a/4 transition, the direct land-sea correlation of the core MD04-2845, located at 45°N in the northern subtropical Atlantic, shows that the increase in ice volume at orbital scale was contemporaneous with a progressive cooling in western Europe, as indicated by the replacement of temperate forest by boreal forest (mainly *Abies* and *Picea*). The simultaneous stabilization of warm SST in the Bay of Biscay demonstrates for the first time a long-term increase of the thermal warm sea-cold land gradient toward the MIS 4 glacial (Sánchez Goñi et al., 2013) (**Figure 11**). At sub-orbital scale, a decoupling with cooling on land synchronous with warming in SST is furthermore observed (**Figure 12**). Three successive episodes of strong land-sea thermal gradient and another three with weak gradients occurred during the MIS 5a/4 transition, superimposed on the long-term increasing trend in the thermal gradient (Sánchez Goñi et al., 2013).

The strong land-sea thermal gradients coincided with the North Atlantic cold events C20, C19, and C18′ marked by moderate iceberg discharges in the western side of the North Atlantic (McManus et al., 1994) and related to Greenland cold episodes GS 21, GS 20, and GS 19 (Rasmussen et al., 2014) while the weak gradients coincided with the warm episodes in the North Atlantic and Greenland, GI 20, GI 19, and GI 18. Whereas the events C20, C19, and C18′ were cold in the subpolar gyre and cold conditions installed in western Europe, the northern subtropical gyre remained warm. Increased snowfall in northern Europe and subsequent ice growth resulted from the high rates of moisture production resulting from the abovementioned strong land-sea thermal gradient and its transport by northward tracking storms. Southward displacement of tundra by 10° in latitude during cold phases C20, C19 and C18′, as suggested by boreal forest colonization of western Europe, probably amplified ice growth owing to the increase in surface albedo (Crucifix and Loutre, 2002). Weak gradients slowed down the process but still allowed ice accumulation. The marine and pollen palaeoclimatic records from core MD99-2331, off north-western Iberia, show the same orbital and sub-orbital increases in the land-sea thermal gradient, excluding during C19 (**Figure 12**). In contrast, further south, in the south-western Iberian margin, the thermal gradient remained weak throughout the MIS 5a/4 transition. In summary, the direct comparison between marine and terrestrial records from the European margin demonstrates for the first time a long-term increase in the thermal gradient between the cold air and warmer sea, and three short intervals of even more pronounced thermal gradients during the last entering in glaciation. This synergy between orbital and millennial-scale variability provided a substantial source of moisture that was transported, through northward-tracking storms, to feed ice sheets in colder Greenland, northern Europe, and the Arctic.

### The Origin of the Millennial-Scale Variability and the Implication for the Ice Ages

The origin of the short-lived climate changes is still a subject of debate. On the one hand, some authors propose that high-latitude processes such as iceberg discharges and changes in the AMOC would have forced the rapid climate changes observed in the mid and low latitudes of the North Atlantic Ocean throughout the Pleistocene (Ganopolski and Rahmstorf, 2001). On the other hand, other authors put forward a low-latitude forcing mechanism via the harmonics of precession. Actually, the tropical latitudes receive over the course of the year a daily irradiance characterized by a double maximum that originates in the equatorial insolation cyclicities at 5,500 and 11,000 years (Berger et al., 2006). A direct consequence of this process would be a larger latitudinal thermal gradient and the enhanced transport of warmth and moisture by either atmospheric (westerlies) or oceanic circulation (subtropical gyre) from equatorial to high latitudes in the North Atlantic (Berger et al., 2006).

**FIGURE 13:**
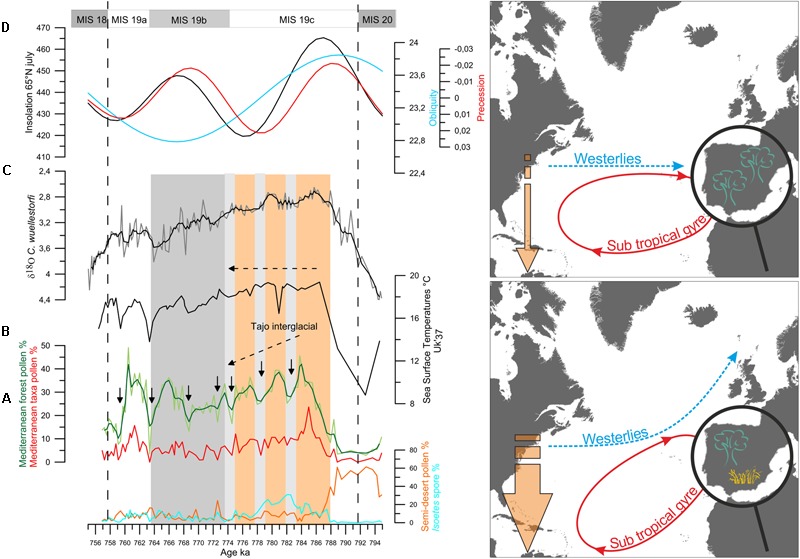
(Left) Vegetation changes versus the δ^18^O_b_ record from Site U1385 for MIS 19. **(A)** Percentages of *Isoetes* spores (cyan) and of semi-desert plants (*Ephedra distachya*-type, *E. fragilis*-type, Chenopodiaceae that now is included in the Amaranthaceae pollen morphotype, *Artemisia*) in orange, pollen percentages of the Mediterranean taxa (evergreen *Quercus, Olea, Pistacia, Phillyrea, Cistus*) in red and Mediterranan forest in light green (mainly deciduous *Quercus*, and Mediterranean taxa). Dark green curve: three-point weigthed-average smoothing, **(B)** Uk′37-based SST record, **(C)** δ^18^O record from benthic foraminifer (δ^18^Ob) *Cibicidoides wuellerstorfi*, Black curve: three-point weighted-average smoothing, **(D)** changes in insolation at 65°N in July (in black), in the precession index (e^∗^sin ω) (in red) and obliquity (light blue) (Berger and Loutre, 1991). Orange and gray vertical panels indicate the Tajo interglacial and the MIS 19b, respectively. Long dashed lines indicate the MIS 19 boundaries. Black arrows indicate Mediterranean forest contractions. Light gray bands indicate the intervals of relatively low Mediterranean forest cover that coincide with heavier benthic δ^18^O values. (Right) Schematic representation of the vegetation in the Iberian Peninsula, atmospheric and surface ocean circulations and temperature gradient in the North Atlantic during millennial events of MIS 19c and MIS 11c. Adapted from Desprat et al. (2017).

This low latitude forcing has also been suggested during periods of low eccentricity, when precession changes are muted, such as the last 45,000 years and MIS 19, centered at 800,000 years ago, based only on paleoceanographic evidence (e.g., McIntyre and Molfino, 1996; Ferretti et al., 2015). Our recent land-sea direct correlation studies at the IODP Site U1385 (south-western Iberian margin) have provided firm evidence on the transport of energy from equatorial regions to the high latitudes by means of the westerlies during the MIS 19 and MIS 11, the astronomically closest analogs to the present interglacial. During MIS 19 and MIS 11, Mediterranean forest pollen percentages indicate two and three long-term major Mediterranean forest expansions, respectively, following maxima in insolation (**Figure 13** for MIS 19 as an example). These major forest episodes point to the occurrence of a well-established Mediterranean climate, with higher winter precipitations and warmer and drier summers. Forest expansion in south-western Iberia therefore indicates that the prevailing zonal configuration of the westerlies during these interglacials brought precipitation to this region (Oliveira et al., 2016; Sánchez Goñi et al., 2016b). During MIS 19c and MIS 11c, a long-term decrease in the Mediterranean forest cover indicating a progressive cooling and drying trend contrasts with the SST record showing quite stable and warm conditions (around 18°C) in the subtropical gyre. During these intervals, freshwater input is not detected on this margin and the progressive increase in the local benthic foraminifer δ^13^C indicates good deep water ventilation (Oliveira et al., 2016; Sánchez Goñi et al., 2016b). These long-term trends were punctuated by major contractions of the Mediterranean forest coeval with increases in Mediterranean semi-desert plants, which may indicate intervals when the westerlies were slightly deflected toward the north with a subsequently reduced influence in south-western Iberia. Repeated meridional shifts in the westerlies during MIS 19c and MIS 11c would have implied successive large masses of warm and moist air reaching the high latitudes of the North Atlantic via the western boundary current of the North Atlantic subtropical gyre. At sub-orbital time scales a decoupling also occurred during MIS 19c and MIS 11c with several drying and cooling events on land concomitant with stable warm SST.

The direct comparison of marine and pollen proxies shows therefore a clear air-sea decoupling during MIS 19c and MIS 11c at orbital and millennial time-scales. In contrast, the other cool and dry events during MIS 19b-19a and MIS 11b-11a were contemporaneous with SST cooling in the subtropical gyre and IRD deposition at in the subpolar gyre (IODP Site U1314 and ODP Site 980), which evidences the input of large amounts of icebergs in the subpolar North Atlantic (Oppo et al., 1998; Alonso-Garcia et al., 2011). Interestingly, the atmospheric cool and dry events during MIS 19c and MIS 11c were not associated with freshwater pulses in either the subtropical or subpolar gyres indicating that the cause of these events was not primarily related to high latitude ice-sheet dynamics. Fourier spectral analysis applied to the pollen and marine records, excluding the benthic foraminifer δ^18^O record, show dominant 5,000- and 10,000-year cyclicities for MIS 19 and MIS 11, likely related with the fourth and second harmonics of precession, respectively (Oliveira et al., 2016; Sánchez Goñi et al., 2016b; Oliveira, 2017). For MIS 19c, the comparison of the Mediterranean forest pollen record and its 5-kyr bandpass filter output curve with the variations in the largest amplitude of the seasonal cycle reconstructed at the equator shows good correspondence between the two records although the magnitude of changes was larger during MIS 19b-19a than during MIS 19c (Sánchez Goñi et al., 2016b). This good correspondence gives support to the low latitude origin of the observed repeated shift of the westerlies in southwestern Iberia at 37°N likely related to the harmonics of precession.

The stronger forest contractions during MIS 19b-19a and MIS 11b-11a would be explained by the regional SST cooling linked to the freshwater fluxes arriving at the subpolar gyre and reducing the AMOC. These data show once more that cold and dry episodes occurred independently of the amount of ice volume (Oppo et al., 1998; Desprat et al., 2009). In contrast, their intensity and duration are modulated by positive feedback mechanisms on the AMOC associated with ice dynamics and, particularly, with freshwater pulses from iceberg discharges and melting (Barker et al., 2015; Oliveira et al., 2016).

During the MIS 19c/19b transition the three cold land-warm sea decoupling events coincide with three increases in the benthic δ^18^O values concomitant with decreases in sea level. This observation suggests that for a period of low amplitude changes in insolation a tropically-driven strong warm sea-cold land contrast at millennial-timescale in the western European margin likely contributed to the increase of moisture. Similarly to that described previously for the MIS 5a/4 transition, these repeated millennial-scale episodes of moisture increase fed the ice caps through northward storm tracks and triggered the onset of the successive glacial period, at around 774,000 years ago.

## Conclusion

Pollen analysis from deep-sea sedimentary sequences constitutes a powerful tool for reconstructing regional vegetation and climate changes that, in turn, influenced the global climate. One of the main advantages of pollen records from marine sediment sequences is that they allow direct correlations of climate change over land, in the ocean and in the ice domain, with minimum chronological uncertainty. This is because marine proxies (e.g., δ^18^O from foraminifer, dinoflagellate cysts, IRD) and terrestrial tracers (e.g., pollen) can be analyzed from the same sediment sample so ocean-ice-atmosphere paleoreadings can be obtained for the same time slice. A second crucial advantage is that besides pollen records, very few other palaeoclimate markers exist that are sensitive to the seasonality of temperature and precipitation. The pollen richness of marine sediments allows the quantitative estimation of temperatures, precipitation and, more importantly, their seasonality, one of the key parameters for understanding climate change. However, further research is necessary for improving the quantification of key climatic parameters, particularly seasonality in subtropical and tropical regions, and of vegetation cover and composition to better estimate albedo changes and their impact on global climate.

This overview has shown that European vegetation and climate responded to long-term and shorter-term climate changes. There was a dynamic equilibrium between vegetation and climate for short periods of forcing such as the D-O cycles and HEs that were similar in magnitude and velocity to the present-day global warming. However, the magnitude of the millennial-scale changes of the last glacial period was regionally-specific. Regions below 40°N appear to have been imprinted by precession, through changes in the amplitude of seasonal contrasts and particularly winter precipitation, while the amplitude of warming in northern regions seems to have been modulated by obliquity that determines the annual temperatures. Within GS and GI, particularly during HS 4, GI 12 and GI 8, a decoupling between high and lower latitudes is observed, a decoupling that has also been identified for the amplitude of the European interglacials. In the mid-latitude regions above 40°N the forest expansion maxima, i.e., the warmest peaks, of the last five terrestrial interglacials is similar while below 40°N their magnitude differed. In this latter region, the main factor for forest expansion is winter precipitation, and this climatic parameter depends on precession.

The overview presented here also suggests that millennial scale climatic variability likely played an important role in glacial inception whatever the boundary climatic conditions were. During one of the largest and fastest ice growth phases at the MIS 5a/4 transition, the millennial variability associated with moderate iceberg discharges in the western North Atlantic Ocean pushed the warm and saline Gulf Stream toward the European margin and created optimal conditions to develop repeated and pronounced air-sea thermal contrasts in this region. The synergy between low and high frequency climate changes amplified moisture production and snow fall in the high latitudes of the Northern Hemisphere. Within interglacials MIS 11 and MIS 19, the cyclicity of the harmonics of precession, repeatedly warming the low latitudes and increasing the latitudinal thermal gradient, created similar pronounced increases of the air-sea thermal gradient in the European margin that favored moisture production and the onset of ice growth. The theoretical model proposed here, and therefore the physical mechanisms involving rapid changes in the westerlies, in the direction of the subtropical gyre and in the air-sea thermal contrast superimposed to the long-term change, needs to be evaluated by model experiments. In particular, some authors (e.g., Crucifix et al., 2016) propose the integration of stochastic models in the Earth Model of Intermediate Complexity with the aim to analyze the effect of the rapid, millennial scale, variability on the long-term glacial-interglacial climate changes.

## Author Contributions

MFSG designed the synthesis and wrote the manuscript. All the co-authors contributed to the writing of the manuscript.

## Conflict of Interest Statement

The authors declare that the research was conducted in the absence of any commercial or financial relationships that could be construed as a potential conflict of interest.

## Supplementary Material

TABLE S1 List of the 129 deep-sea pollen records distributed over the world.
